# Editorial of Special Issue “Combining Sensors and Multibody Models for Applications in Vehicles, Machines, Robots and Humans”

**DOI:** 10.3390/s21196345

**Published:** 2021-09-23

**Authors:** Javier Cuadrado, Miguel Á. Naya

**Affiliations:** Laboratory of Mechanical Engineering, University of La Coruña, 15403 Ferrol, Spain; miguel.naya@udc.es

The combination of physical sensors and computational models to provide additional information about system states, inputs and/or parameters, in what is known as virtual sensing, is becoming more and more popular in many sectors, such as the automotive, aeronautics, aerospatial, railway, machinery, robotics and human biomechanics sectors. While, in many cases, control-oriented models, which are generally simple, are the best choice, multibody models, which can be much more detailed, may have applications, for example, during the design stage of a new product.

This Special Issue sought to attract works dealing with the many challenges that must be overcome when developing multibody-based virtual sensors. These challenges include the selection of the fusion algorithm and its parameters, the coupling or the independence between the fusion algorithm and the multibody formulation, the magnitudes to be estimated, the stability and accuracy of the adopted solution, optimization of the computational cost, real-time issues and implementation on embedded hardware. Studies of the application of multibody-based virtual sensors to, for example, vehicles, heavy machinery, mobile or humanoid robots, assistive orthotic and prosthetic devices or the measurement and analysis of human movement were also welcome.

In the next paragraphs, a brief description of the content of each contribution forming the Special Issue is provided.

The first work [1] focuses on the measurement of human motion and employs an extended Kalman filter to carry out the optical motion capture, the problem being to adjust the filtering level of the algorithm, for which the accelerometers contained in inertial measurement units are used.

The second work [2] addresses the measurement of railway track geometric irregularities by means of a system included on board, composed of a kinematic model of the track and a set of sensors.

The objective of the third work [3] is to avoid the danger of reaching singular positions in a parallel robot for rehabilitation. For this purpose, a control system is developed based on computer vision and the multibody model of the robot.

The fourth work [4] is not centered on a particular field of application, but on the general problem of state and input estimation, its contribution being a method to extract the matrices required for a discrete Kalman filter which provides stable and accurate results, demonstrated in a slider–crank mechanism.

The fifth work [5] foresees naval applications in the future, although it tackles the general problem of state, input and parameter estimation, proposing that the model equations are based on Pontryagin’s treatment of Hamiltonian systems, and that feedback is used to re-initialize the initial values of a reformulated two-point boundary value problem.

The sixth work [6] deals with human-in-the-loop applications with haptic feedback, providing examples in the automotive and musical fields. Sensors are used to characterize and validate the multibody model, to measure the system kinematics and dynamics within the human-in-the-loop process, and to validate the haptic device behavior.

The field of application of the seventh work [7] is machinery. More specifically, the work seeks to determine the characteristic curves of a directional control valve in a hydraulic system. For this purpose, it employs an augmented discrete extended Kalman filter based on a multibody model of the mechanism and the lumped fluid theory for a fluid power system, along with a curve-fitting method to describe the characteristic curves of the valve.

The eighth work [8] looks at the general problem of state and force estimation, considering the automotive field as a clear target of application. The authors apply an adaptive method to estimate the noise covariance matrices of a state and input estimator based on multibody models.

The ninth work [9] intends to estimate the sideslip angle in road vehicles, and proposes an alternative to traditional methods of state estimation by representing the problem as a probabilistic graphical model which can be optimized by several methods.

The tenth work [10] is a spatial application consisting of the design of a six-legged integrated lander and rover for repetitive lunar landings. The possibilities of successful landing despite the failure of some integrated drive units of the system are explored, assuming that at least three legs of the hexapod are operative.

In summary, it can be seen that the papers gathered in the Special Issue contributed either by proposing solutions to the general problem of state, input and/or parameter estimation [4,5,7,8,9], and/or by suggesting applications of the combined use of sensors and multibody models to different fields, such as automotive [6,8,9], railway [2], naval [5], spatial [10], machinery [4,7], robotics [3], biomechanics [1] and music [6] applications, thus showing the theoretical challenges and practical interest of this research topic.

Finally, we wish to thank the authors, reviewers and journal staff for their commitment and effort, which made it possible to complete this Special Issue on time.
